# Effects of Different Natural Factors on Rheological Properties of SBS Modified Asphalt

**DOI:** 10.3390/ma15165628

**Published:** 2022-08-16

**Authors:** Shanglin Song, Meichen Liang, Linbing Wang, Dongna Li, Meng Guo, Luchun Yan, Xingjun Zhang, Weixun Ding

**Affiliations:** 1 National Center for Materials Service Safety, University of Science and Technology Beijing, Beijing 100083, China; 2 Scientific Observation and Research Base of Transport Industry of Long Term Performance of Highway Infrastructure in Northwest Cold and Arid Regions, Dunhuang 736200, China; 3 Gansu Henglu Traffic Survey and Design Institute Co., Ltd., Lanzhou 730070, China; 4 The Key Laboratory of Urban Security and Disaster Engineering of Ministry of Education, Beijing University of Technology, Beijing 100124, China; 5 The College of Mechanical and Electrical Engineering, Lanzhou Jiaotong University, Lanzhou 730070, China; 6 Gansu Province Highway Traffic Construction Group Co., Ltd., Lanzhou 730030, China

**Keywords:** road engineering, SBS-modified asphalt, natural aging, rheological properties, aging contribution index

## Abstract

Typical climatic environments such as UV radiation, high temperature and strong wind in cold and arid regions have a significant effect on asphalt aging. The intent of this work is to reveal the evolution law of natural aging of SBS-modified asphalt under the complex adverse climate environment in cold and arid regions. Furthermore, the contribution rate of various environmental factors of natural aging of asphalt in cold and arid regions was analyzed. Based on rheological parameters, this paper characterized the influence of natural aging on the viscoelastic properties, rutting resistance at a high temperature, fatigue resistance and cracking resistance at a low temperature of SBS-modified asphalt. The evolution law of natural aging performance of SBS-modified asphalt was revealed. A quantitative evaluation index (*CI*i) of natural aging contribution rate of asphalt was put forward and the contribution rate of various environmental factors to asphalt natural aging was analyzed. The results showed that the effects of simulated aging and natural aging on asphalt properties were similar. After aging, the viscoelastic properties of asphalt were deteriorated, and the risk of fatigue cracking and low temperature cracking was increased. It also enhanced the deformation resistance of asphalt and increased the rutting resistance at high temperature. The aging contribution index *CI*i obtained based on rheological parameters such as complex modulus and rutting factor could directly reflect the influence of different natural factors on the performance of asphalt during aging. Among them, the effect of thermal oxygen was more obvious on the natural aging of SBS-modified asphalt.

## 1. Introduction

Asphalt pavement is exposed to a complex environment for a long time. Affected by external factors such as high temperature, sunshine, rain and oxygen, it is easy to cause cracking, loose, rutting and other diseases, and its service life is much lower than the design life [1,2]. However, the asphalt-aging factors considered are still concentrated on thermal oxygen, UV, water and other factors, which are relatively single, and most of them also use laboratory simulation methods, which cannot reflect the service conditions of asphalt pavement in the real environment [3,4]. The asphalt samples at different aging stages are mainly obtained through accelerated simulation in the laboratory. Standard specification of asphalt always applied short-term aging to simulate the effect of high temperature and oxygen on asphalt in the process of mixture mixing, transportation and paving. The experimental conditions are selected as 163 °C and 75 min. Long-term aging was selected to simulate the aging of 5 years of pavement service. Long-term aging experimental conditions are selected as 2.1 Mpa pressure and 100 °C. The aging of asphalt by UV and oxygen is simulated by UV irradiation of high-pressure mercury lamp [5,6]. Laboratory-accelerated simulated aging test generally requires some kind of forced aging of asphalt. It will magnify natural environmental factors, and its aging conditions generally more severe than the outside effect, to obtain a large amount of data in a short time. Simulated environment factor is relatively single, which did not consider the interaction between multiple factors and the coupling effect. It also ignored the influence of rainfall, acid and alkali and various kinds of chemical medium, simulation results and the actual service environment of asphalt pavement is having a bigger difference [7]. In addition, the coupled action of different environmental factors impact on the asphalt performance has a bigger difference. However, there is no evaluation method for the contribution rate of environmental factors to asphalt aging, and the proportion of the influence of various environmental factors on asphalt-aging degree is not clear. It also reduces the prediction accuracy of the different regions of the asphalt performance, asphalt pavement protection in different environmental areas cannot be targeted.

In order to accelerate the simulation of asphalt aging and analyze the influence of single factor or multiple-specific factors on asphalt aging, an indoor-simulated aging test was developed. It is also the main mean to study the aging behavior of asphalt pavement in the domestic and overseas. Sun et al. [8,9] conducted coupling aging tests on asphalt under different temperatures and UV light intensity. It was found that the light intensity had a greater impact on the softening point of asphalt, but a minor impact on the viscosity. Referring to the actual UV light intensity of the asphalt pavement in the field, Fen et al. [10,11,12] made a strong UV aging box, taking softening point and ductility as performance indexes, the change law of asphalt performance before and after aging was determined, the results showed that the strong UV radiation will cause serious aging of asphalt. Qian [13] believed that the role of rain can easily lead to the loss of soluble substances in asphalt, thus leading to the aging of asphalt. Zhang et al. [14] studied the influence of precipitation on the components of asphalt binder. It was found that the organic acid salt with low cost and high grade in asphalt will act as emulsifier in the presence of water. As a result, the free energy of the asphalt surface decreases the dissolved asphalt components. Lu et al. [15] proposed that after thermal oxygen aging (rolling thin-film oven test, RTFOT; thin-film oven test, TFOT; and pressurized aging vessel, PAV), the asphalt viscosity increased and the asphalt hardened. The rutting resistance of asphalt at high temperature enhanced and the cracking resistance at low temperature weakened. As a result, some of the components of asphalt had strong sensitivity to temperature, especially when the temperature changed suddenly, the internal structure of asphalt also changed, so the aging behavior was also accelerated [16,17].

Asphalt at room temperature will also provoked aging, which is generally slow. However, with the increase in temperature, the aging rate increased exponentially. In conclusion, many scholars have carried out a lot of work on asphalt aging. In terms of a test, the most widely used was the indoor heating aging test [18,19]. In recent years, light, water, oxygen and other factors were also gradually considered in the asphalt-aging test, but as a result of the multi-factor coupling aging test, it produces less data. There is a lack of real environment asphalt-aging data accumulation, the mechanisms of the aging process of the asphalt material has no definite conclusion, and it does not have a suitable asphalt-aging evaluation system and evaluation standard. Particularly, in cold and arid regions, where the climate is characterized by large temperature difference, extreme drought desert, strong winds, as well as strong UV radiation, the natural aging mechanism and evolution of asphalt still need further research [20,21].

In this paper, SBS-modified asphalt was exposed to natural aging under the severe climatic environment of strong UV, large temperature difference and extremely dry desert in the cold and arid regions of Northwest China. Based on rheological parameters, the aging law of SBS-modified asphalt in the outdoor environment was studied and the contribution rate of different natural aging environmental factors was quantitatively analyzed. The test results will help reveal the aging mechanism of asphalt in different climatic environments and improve the matching degree between indoor-simulated aging parameters and natural aging. It also can develop an accelerated aging simulation test device in cold and dry areas, which will make a solid theoretical foundation for further research and development of durable-modified asphalt suitable for different climatic regions. A comprehensive evaluation system for asphalt aging and decay will be improved, which has a great significance to the development of durable asphalt pavements in Northwest China.

## 2. Materials and Test Methods

### 2.1. Materials

As a kind of asphalt, SBS-modified asphalt has been widely used in asphalt pavement construction. Compared with unmodified asphalt, it has good high-temperature rutting resistance and low-temperature fatigue resistance [18]. The basic performance indexes of the Styrene–Butadiene–Styrene (SBS) modified asphalt were shown in Table 1. The asphalt with a penetration of 6.6 mm and ductility at 5 °C of 42 cm is commonly used in China, meeting the requirements of technical indicators.

### 2.2. Preparation of Aged Asphalt

#### 2.2.1. Laboratory Aging

The short-term aging asphalt samples and long-term aging asphalt samples of SBS-modified asphalt was got in the laboratory. The short-term aging samples were obtained by the rolling thin-film oven test (JTG E 200-2011/T 0610). The asphalt was put into the rolling thin-film oven, then the aging temperature was set at 163 °C and it was aged for 75 min to obtain short-term aging asphalt. Pressurized aging vessel was used for long-term aging test. Asphalt samples used for PAV test were after RTFOT aging. In total, 10 samples could be placed in the box at one time. The aging temperature was set to 100 °C, the box was pressurized by 2.1 MPa and the aging time was 20 h.

#### 2.2.2. Outdoor Natural Aging

**Figure 1:**
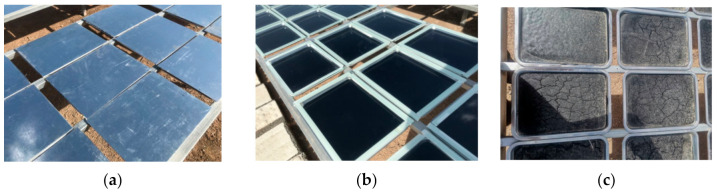
Natural aging of asphalt in the field. (**a**) Thermal oxygen aging; (**b**) Thermal oxygen + UV aging; (**c**) All-weather aging.

**Figure 2:**
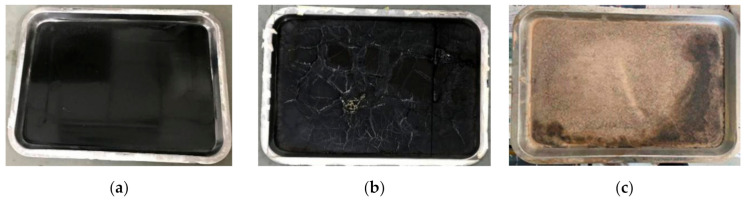
SBS-modified asphalt natural aging samples. (**a**) Thermal oxygen aging; (**b**) Thermal oxygen + UV aging; (**c**) All-weather aging.

In order to better carry out the comparative analysis of indoor and outdoor asphalt aging, this test relied on Scientific Observation and Research Base carried out research on natural exposure aging of asphalt. This base is called the Scientific Observation and Research Base of Transport Industry of Long Term Performance of Highway Infrastructure in Northwest Cold and Arid Regions. Three exposure methods were adopted for the test and the unaged asphalt samples were put on the stainless-steel shelf of the base. Thickness of the asphalt sample was set to 3.18 mm. All samples were exposed to strong UV radiation and large temperature difference in the environment that act on asphalt aging for 1 year (Figure 1). Figure 1a was set as a “control” group to record the thermal oxygen (TO) aging; therefore, the sample was covered by opaque plates to isolate UV radiation, water and dust. Figure 1b was the sample that was covered by transparent plates to make asphalt undergoing thermal oxygen aging and UV radiation, while isolated from water and dust. Figure 1c is the sample that was directly exposed to environment, so that the asphalt samples were aged under the comprehensive effect of the natural environment. Observation and analysis of their properties were carried out after a year (Figure 2).

### 2.3. Study on Rheological Properties

In this study, a dynamic shear rheometer DHR-2 produced by TA Instruments company (New Castle, DE, USA) was selected to analyze the rheological properties of asphalt. The angular frequency range was set to 100~0.1 rad/s, and the temperature was set to 35 °C, 45 °C and 55 °C. The strain control mode was selected and set to 1%. Based on the time–temperature equivalence principle (TTPS) [22], the complex modulus of asphalt obtained at different temperatures could be shift to a reference temperature by changing its loading frequency to form a single curve, which is called the master curve. In this study, the least square method was applied to form the master curve of the complex modulus. In addition to the complex modulus master curve, the rutting factor, G*/sinδ, which varies with temperature, was also applied to characterize the rutting resistance of asphalt at a high temperature. Glover Rowe (G-R) parameters were calculated according to Equation (1) to characterize the fatigue resistance of asphalt at a medium temperature [23,24]. Additionally, to characterize the cracking resistance of asphalt at a low temperature, the stiffness modulus (S) and creep rate (m) were obtained at −6 °C, −12 °C and −18 °C by bending the creep stiffness of asphalt (BBR). Stiffness modulus is the reciprocal of creep stiffness of asphalt at different times. The creep rate is the absolute value of the logarithmic slope of creep stiffness and the logarithm of the time curve at 60 s. The stiffness modulus and creep rate of asphalt at −6 °C, −12 °C and −18 °C under a loading time of 60 s were measured, which characterized the low-temperature cracking resistance of different asphalts.

(1) $$\mathrm{GR}=\frac{\left|G\right|\cdot {\left(\cos\delta \right)}^{2}}{\sin\delta }$$

where G* is the complex modulus, kPa, and δ is the phase angles, (rad).

## 3. Results and Discussion

### 3.1. Analysis of Asphalt Natural Aging Performance

#### 3.1.1. Natural Aging Analysis of Asphalt Based on Complex Modulus

**Figure 3:**
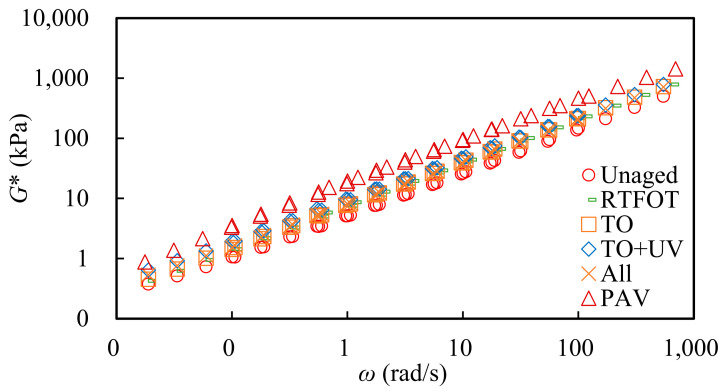
Master curve of complex modulus of SBS-modified asphalt before and after aging.

The influence of thermal oxygen aging, thermal oxygen + UV aging and comprehensive nature aging on viscoelastic properties of SBS-modified asphalt could be evaluated by the shear complex modulus. According to TTPS, the complex modulus curve at different temperatures could be parallel shifted to obtain the complex modulus master curve at a reference temperature. In this study, 45 °C was selected to be the reference temperature, and the complex modulus master curve of SBS-modified asphalt under different ageing conditions is shown in Figure 3.

From Figure 3, it can be seen that the natural aging of the three methods had a great impact on the modulus of SBS-modified asphalt. It was concluded that the natural aging had a large reduction range for viscoelastic properties of SBS-modified asphalt. In comparison, the natural aging of the three methods improved the complex modulus of asphalt, but the growth degree was similar. Among them, the thermal oxygen + UV aging had a greater impact on asphalt than the all-weather aging and thermal oxygen aging. At low frequency (high temperature), the complex modulus of asphalt after aging of the three methods increased relatively greatly, and the order was: thermal oxygen aging + UV aging > all-weather aging > thermal oxygen aging. The three kinds of natural aging asphalt were almost similar to the short-term aging asphalt in the laboratory at high frequency (low temperature).

#### 3.1.2. Asphalt Natural Aging Analysis Based on Rutting Factor

**Figure 4:**
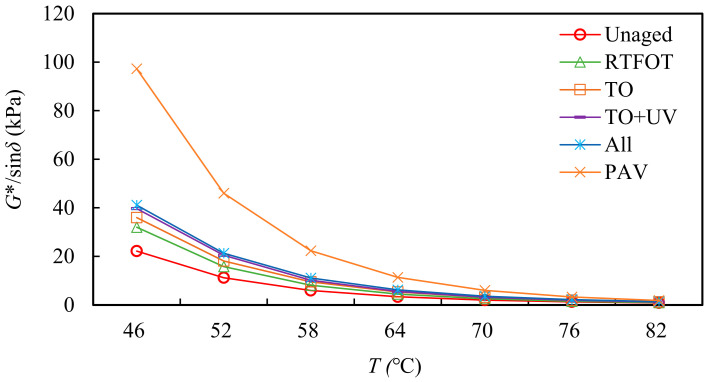
Curve of rutting factor with temperature before and after the aging of SBS-modified asphalt.

Rutting factor (G*/sinδ) was regarded as a rutting resistance indicator that could evaluate high-temperature performance of asphalt. In this study, the influence of thermal oxygen, UV and other environmental factors on asphalt was analyzed based on their rutting factor. Additionally, the rutting factor–temperature curves of different aging conditions are shown in Figure 4.

It can be seen from Figure 4 that the high-temperature rutting resistance and viscoelastic properties show the same law, that the asphalt had an obvious change range before and after natural aging. In comparison, the influence of all-weather aging and thermal oxygen + UV aging on asphalt was similar, and the influence range was larger. According to the rutting factor, the order was: all-weather aging > thermal oxygen + UV aging > thermal oxygen aging. Combined with the changes in viscoelastic properties of asphalt before and after aging, it can be considered that the addition of UV, wind, dust and other factors had a great impact on the high-temperature performance of asphalt, in particular, UV radiation had a great impact on the aging degree of asphalt.

#### 3.1.3. Natural Aging Analysis of Asphalt Based on G-R Parameters

**Figure 5:**
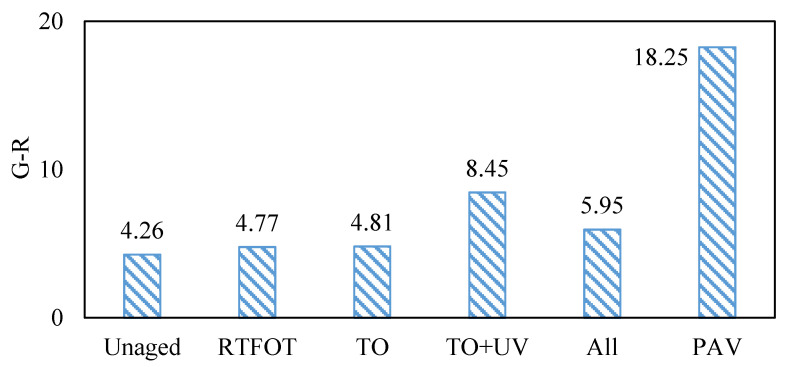
G-R parameters of SBS-modified asphalt before and after aging.

Glover et al. [23] found the rheological parameter G′(η′/G′) at 15 °C and 0.005 rad/s has a good correlation with the results of 15 °C ductility test, which can be used to characterize the fatigue performance of asphalt. Rowe et al. [24] believed that G′(η′/G′) can be further simplified by: η′ = G′′/ω and tanδ = G′′/G′ can be obtained η′/G′ = (1/ω) × (G′′/G′) = tanδ/ω, then G′/(η′/G′) = G′ × ω/tanδ, simplified to G′ × ω/tanδ = (|G*| × (cosδ)^2^ × ω)/sinδ, the frequency is a fixed value of 0.005 rad/s, which can be omitted to obtain G* and δ function G*(cosδ)^2^/sinδ, it can effectively characterize the fatigue cracking resistance of asphalt. The G-R parameters of base asphalt undergoing different aging methods are shown in the following figure. The G-R parameters of SBS-modified asphalt undergoing different aging methods are shown in Figure 5.

It can be seen from Figure 5 that thermal oxygen aging had little effect on the fatigue resistance of SBS-modified asphalt, but all-weather aging and thermal oxygen + UV aging had greatly increased the G-R parameters of asphalt, resulting in an increase in the risk of fatigue cracking. The influence range of all-weather aging was less than the thermal oxygen + UV aging, which was together with the dust adsorb on the all-weather aging surface, hindering the aging of asphalt by oxygen and UV.

#### 3.1.4. Natural Aging Analysis of Asphalt Based on Stiffness Modulus

**Figure 6:**
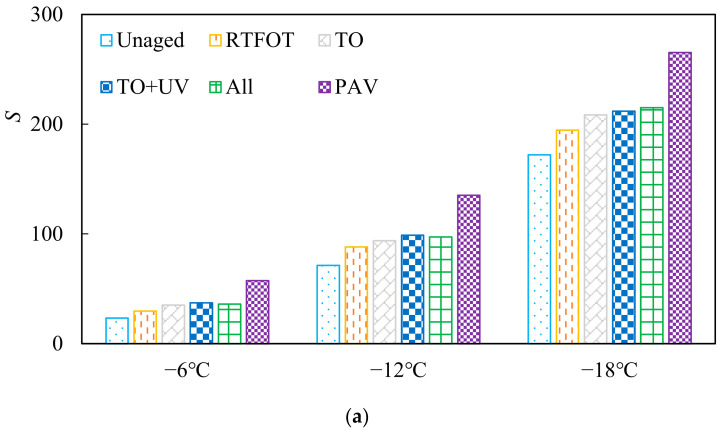

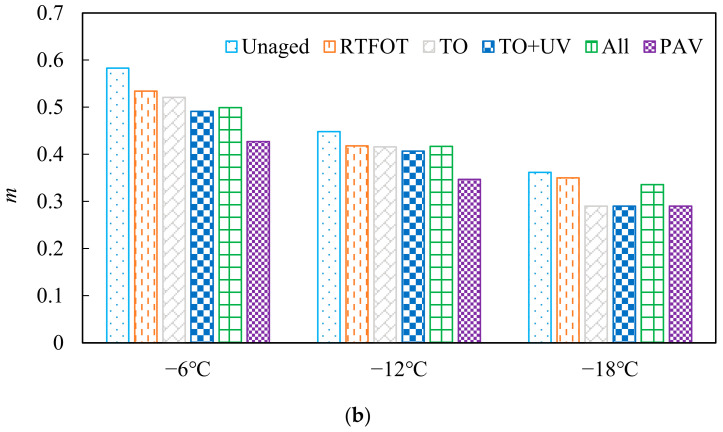
The stiffness modulus and creep rate of asphalt binder before and after aging: (**a**) stiffness modulus *S*; (**b**) creep rate *m*.

Asphalt will harden under the action of low temperature, which is characterized by Hooke elastomer, and deformation will occur at the moment of stress application. Stiffness modulus S was considered to be a reliable index to reflect the flexibility of asphalt at low temperature. The greater the stiffness modulus of asphalt, the smaller the allowable strain. The creep rate was a supplemental indicator, which reflects the deformation rate of asphalt at a low temperature and certain temperature stress. The larger the m value is, the faster the asphalt deformation changes, and the better the low-temperature cracking resistance is. The low-temperature performance test results of SBS-modified asphalt with different aging methods are shown in Figure 6.

It can be seen from Figure 6 that the stiffness modulus of different-aged SBS-modified asphalt did not exceed 300 mpa, indicating that the aged SBS-modified asphalt still met the specification requirements, and the SBS modifier played a role in delaying the increase in low-temperature risk of asphalt during the aging process. At three temperatures, it can be found that three kinds of natural aging significantly increased the reduction in the asphalt S value, but at −6 °C and −12 °C, the impact of all-weather aging on asphalt stiffness modulus was slightly less than that of thermal oxygen + UV aging. This was similar to the G-R parameter law. The dust adsorbed on the all-weather aging surface hindered the contact of oxygen and UV with asphalt.

In conclusion, for SBS-modified asphalt, natural aging for one year had little effect on its aging degree. Among them, thermal oxygen aging had a greater effect on the aging degree of SBS-modified asphalt, but it was still similar to the short-term aging degree. However, SBS-modified asphalt was significantly affected by rain, wind, dust and other effects. The impact of all-weather aging on viscoelastic properties, G-R parameters and low-temperature performance was less than that of thermal oxygen + UV aging. It can be seen that the dust covered on the surface of SBS-modified asphalt reduced its contact area with the external environment to a greater extent and reduced the aging of asphalt by oxygen, UV, etc. Although the degree of natural aging was small, it can still clearly be found that the thermal oxygen and UV radiation deepened the aging degree of SBS-modified asphalt. Temperature change and strong UV are the greatest challenges faced by pavements in Northwest China.

### 3.2. Analysis of Asphalt Natural Factors

The above content mainly studied the influence of natural aging on the properties of SBS-modified asphalt. The evolution law of asphalt natural aging was explored and it was found that there were great differences in the influence of different natural factors on asphalt. However, it was difficult to reflect the influence of various environmental factors on asphalt natural aging through qualitative analysis, due to the small influence of natural aging on asphalt performance in one year. Based on the above test, a quantitative evaluation index for the contribution rate of natural inducements to asphalt natural aging—asphalt natural aging contribution index *CI*i—will be proposed. It was used to quantitatively evaluate the influence of different aging factors on the natural aging of asphalt.

#### 3.2.1. Analysis of Natural Aging Factors Based on Complex Modulus

In order to quantitatively evaluate the influence of three natural factors such as thermal oxygen, UV light, rain, wind and dust on the natural aging degree of asphalt, the contribution index CI_G_ of asphalt natural aging was proposed based on the complex modulus of 45 °C, as shown in Equations (2)–(4). The greater the CI_G_, the greater the influence of the aging inducement on the viscoelastic properties of asphalt.

(2) $${\mathrm{CI}}_{G\;\mathrm{TO}}=\frac{G_{\mathrm{TO}}-G_{\mathrm{unaged}}}{G_{\mathrm{all}\;\mathrm{aging}}-G_{\mathrm{unaged}}}$$

(3) $${\mathrm{CI}}_{G\;\mathrm{UV}}=\frac{G_{\mathrm{TO}+\mathrm{UV}\;\mathrm{aging}}-G_{\mathrm{TO}}}{G_{\mathrm{all}\;\mathrm{aging}}-G_{\mathrm{unaged}}}$$

(4) $${\mathrm{CI}}_{G\;\mathrm{others}}=\frac{G_{\mathrm{all}\;\mathrm{aging}}-G_{\mathrm{TO}+\mathrm{UV}\;\mathrm{aging}}}{G_{\mathrm{all}\;\mathrm{aging}}-G_{\mathrm{unaged}}}$$

where CI_G TO_ is the contribution index of thermal oxygen to asphalt natural aging; CI_G UV_ is the contribution index of UV action to asphalt natural aging; CI_G others_ is the contribution index of other factors to asphalt natural aging; G_TO aging_ is the complex modulus of thermal oxygen aging asphalt at 45 °C, kPa; G_TO+UV aging_ is the complex modulus of thermal oxygen + UV aging asphalt at 45 °C, kPa; G_All aging_ is the complex modulus of all-weather aging asphalt at 45 °C, kPa; and G_unaged_ is the complex modulus of unaged asphalt at 45 °C, kPa.

**Figure 7:**
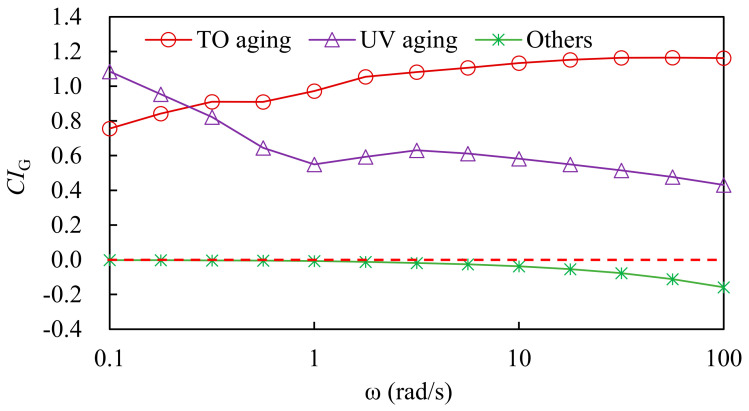
Natural aging contribution index of asphalt based on complex modulus.

According to formulas (2)–(4), the effects of thermal oxygen, UV light and other factors on natural aging were calculated. The results are shown in Figure 7.

As shown in Figure 7, the aging contribution index obtained could directly reflect the impact of different aging factors on the viscoelastic properties of asphalt during natural aging. The effect of thermal oxygen on SBS-modified asphalt was the greatest. With the increase in test frequency, the effect of UV light on SBS-modified asphalt gradually decreased, and the aging contribution index of SBS-modified asphalt fluctuated slightly at different frequencies. At different test frequencies, the influence of thermal oxygen on SBS-modified asphalt fluctuated slightly. The aging contribution index of other factors such as rain, wind and dust on the viscoelastic properties of base asphalt was negative and gradually decreased with the increase in test frequency. This showed that the covering effect of rain, wind and dust on the asphalt surface can reduce the adverse impact of the environment on the viscoelastic properties of asphalt, and other factors such as rain, wind and dust had a certain anti-aging effect.

#### 3.2.2. Analysis of Natural Aging Inducement Based on Rutting Factor

Based on the rutting factor, the contribution index CI_H_ of natural aging of asphalt was proposed. As shown in formulas (5)–(7), the larger the CI_H_, the greater the influence of aging inducement on the high-temperature performance of asphalt.

(5) $${\mathrm{CI}}_{H\;\mathrm{TO}}=\frac{H_{\mathrm{TO}}-H_{\mathrm{unaged}}}{H_{\mathrm{all}\;\mathrm{aging}}-H_{\mathrm{unaged}}}$$

(6) $${\mathrm{CI}}_{H\;\mathrm{UV}}=\frac{H_{\mathrm{TO}+\mathrm{UV}\;\mathrm{aging}}-H_{\mathrm{TO}}}{H_{\mathrm{all}\;\mathrm{aging}}-H_{\mathrm{unaged}}}$$

(7) $${\mathrm{CI}}_{H\;\mathrm{others}}=\frac{H_{\mathrm{all}\;\mathrm{aging}}-H_{\mathrm{TO}+\mathrm{UV}\;\mathrm{aging}}}{H_{\mathrm{all}\;\mathrm{aging}}-H_{\mathrm{unaged}}}$$

where CI_H TO_ is the contribution index of thermal oxygen to asphalt natural aging; CI_H UV_ is the contribution index of UV action to asphalt natural aging; CI_H others_ is the contribution index of other factors to asphalt natural aging; H_TO aging_ is the complex modulus of thermal oxygen aging asphalt at 45 °C, kPa; H_TO+UV aging_ is the rutting factor of thermal oxygen + UV aging asphalt, kPa; H_All aging_ is the rutting factor of all-weather aging asphalt, kPa; and H_unaged_ is the rutting factor of unaged asphalt, kPa.

**Figure 8:**
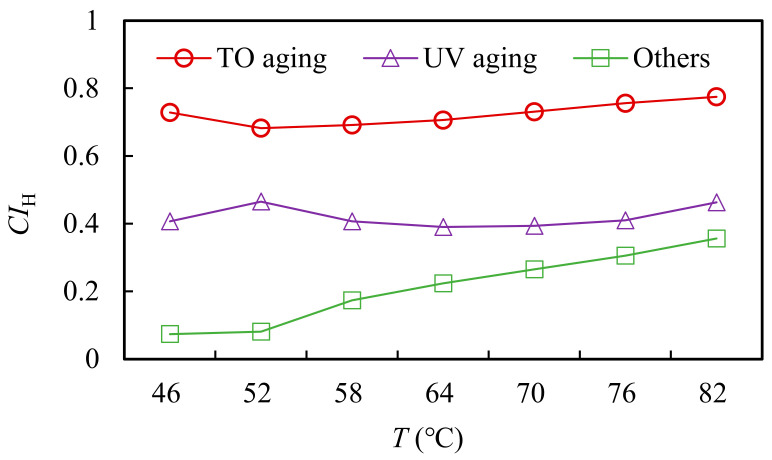
Contribution index of asphalt natural aging based on rutting factor.

According to Equations (5)–(7), the effects of thermal oxygen, UV light and other factors on natural aging were calculated. The results are shown in Figure 8.

Figure 8 reflected the influence of different aging factors on the high-temperature performance of asphalt during the natural aging visually. For SBS-modified asphalt, the effect of thermal oxygen aging on the high-temperature performance of asphalt was more obvious. With the increase in test temperature, the effect of UV light on the matrix asphalt decreased, and the SBS-modified asphalt decreased first and then tended to be flat. It can be seen that the effect of UV light on the performance will decrease under a high temperature. The contribution index of thermal oxygen effect on the high-temperature aging performance of SBS-modified asphalt was lower than that of UV effect, and gradually decreased with the decrease in temperature. Relatively speaking, the effect of thermal oxygen on SBS-modified asphalt was very obvious. At different temperatures, the effect was similar and higher than the effect of UV light. It can be seen that thermal oxygen had a greater impact on the high-temperature performance of SBS asphalt. The aging contribution index of rain, wind, dust and other factors on the high-temperature performance of was SBS-modified asphalt was about 0.36 at 82 °C, which showed an obvious impact.

#### 3.2.3. Analysis of Natural Aging Inducement Based on G-R Parameters

Based on the G-R parameters, the natural aging contribution index CI_R_ of asphalt is proposed, as shown in Equations (8)–(10). The greater the CI_R_, the greater the influence of the aging inducement on the fatigue resistance of asphalt.

(8) $${\mathrm{CI}}_{R\;\mathrm{TO}}=\frac{R_{\mathrm{TO}}-R_{\mathrm{unaged}}}{R_{\mathrm{all}\;\mathrm{aging}}-R_{\mathrm{unaged}}}$$

(9) $${\mathrm{CI}}_{R\;\mathrm{UV}}=\frac{R_{\mathrm{TO}+\mathrm{UV}\;\mathrm{aging}}-R_{\mathrm{TO}}}{R_{\mathrm{all}\;\mathrm{aging}}-R_{\mathrm{unaged}}}$$

(10) $${\mathrm{CI}}_{R\;\mathrm{others}}=\frac{R_{\mathrm{all}\;\mathrm{aging}}-R_{\mathrm{TO}+\mathrm{UV}\;\mathrm{aging}}}{R_{\mathrm{all}\;\mathrm{aging}}-R_{\mathrm{unaged}}}$$

where CI_R TO_ is the contribution index of thermal oxygen to asphalt natural aging; CI_R UV_ is the contribution index of UV action to asphalt natural aging; CI_R others_ is the contribution index of other factors to asphalt natural aging; R_TO aging_ is the complex modulus of thermal oxygen aging asphalt at 45 °C, kPa; R_TO+UV aging_ is the G-R parameters of hot oxygen + UV aging asphalt; R_All aging_ is the G-R parameters of all-weather aging asphalt; and R_unaged_ is the G-R parameters of unaged asphalt.

**Figure 9:**
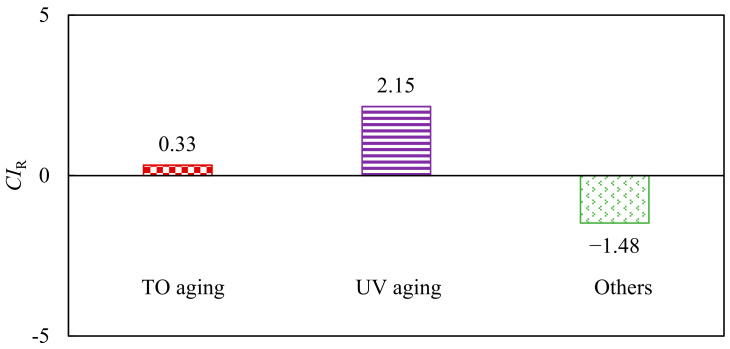
Asphalt natural aging contribution index based on G-R parameters.

According to Equations (8)–(10), the effects of thermal oxygen, UV light and other factors on natural aging were calculated, and the results are shown in Figure 9.

As shown in Figure 9, during the natural aging process of asphalt, the influence of UV action on SBS-modified asphalt was the largest, and the contribution index of rain, wind, dust and other influences was negative. Different from the influence on viscoelastic properties and high-temperature properties, the effect of UV light on the fatigue resistance of SBS-modified asphalt was significantly greater than that of thermal oxygen. The contribution index of rain, wind, dust and other effects to the natural aging of asphalt was negative, indicating that the covering effect of sand blown by the wind accumulation on the asphalt surface could reduce the adverse impact of the environment on the fatigue resistance of asphalt.

#### 3.2.4. Analysis of Natural Aging Inducement Based on Stiffness Modulus

Based on the −12 °C stiffness modulus parameter, the natural aging contribution index *CI*_S_ of asphalt was proposed, as shown in Equations (11)–(13). The greater the *CI*_S_, the greater the influence of the aging inducement on the low-temperature performance of asphalt.

(11) $${\mathrm{CI}}_{S\;\mathrm{TO}}=\frac{S_{\mathrm{TO}}-S_{\mathrm{unaged}}}{S_{\mathrm{all}\;\mathrm{aging}}-S_{\mathrm{unaged}}}$$

(12) $${\mathrm{CI}}_{S\;\mathrm{UV}}=\frac{S_{\mathrm{TO}+\mathrm{UV}\;\mathrm{aging}}-S_{\mathrm{TO}}}{S_{\mathrm{all}\;\mathrm{aging}}-S_{\mathrm{unaged}}}$$

(13) $${\mathrm{CI}}_{S\;\mathrm{others}}=\frac{S_{\mathrm{all}\;\mathrm{aging}}-S_{\mathrm{TO}+\mathrm{UV}\;\mathrm{aging}}}{S_{\mathrm{all}\;\mathrm{aging}}-S_{\mathrm{unaged}}}$$

where CI_S TO_ is the contribution index of thermal oxidation to the natural aging of asphalt; CI_S UV_ is the contribution index of UV action to asphalt natural aging; CI_S others_ is the contribution index of other factors to asphalt natural aging; S_TO aging_ is the stiffness modulus of thermal oxygen-aged asphalt, MPa; S_TO+UV aging_ is the stiffness modulus of thermal oxygen + UV aging asphalt, MPa; S_All aging_ is the stiffness modulus of all-weather aging asphalt, MPa; and S_unaged_ is the stiffness modulus of unaged asphalt, MPa.

**Figure 10:**
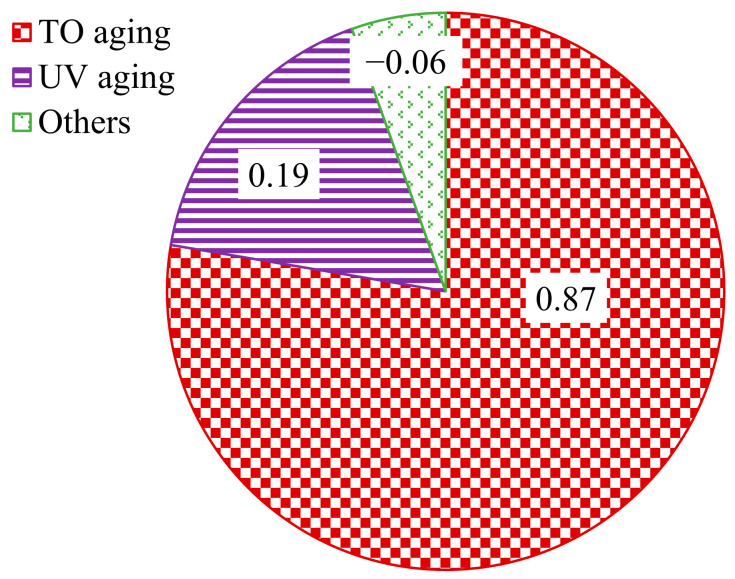
Contribution index of asphalt natural aging based on stiffness modulus.

According to Equations (11)–(13), the effects of thermal oxygen, UV action and other factors on natural aging were calculated. The results are shown in Figure 10.

It can be seen from Figure 10 that the influence of UV on the cracking resistance of SBS-modified asphalt at a low temperature was relatively small, even less than that of thermal oxygen. Thermal oxygen had a great influence on the low-temperature performance of asphalt, and the aging contribution index of SBS-modified asphalt was 0.87. Rain, wind, dust and other factors had no influence on the low-temperature performance of SBS-modified asphalt.

In conclusion, the aging contribution index based on complex modulus, rutting factor, G-R parameter and stiffness modulus could intuitively reflect the influence of different aging factors on the properties of asphalt during its natural aging process. As show in Table 2, thermal oxygen aging had a great influence on the performance of SBS-modified asphalt. Despite the fact that the contribution index of thermal oxygen aging based on G-R parameters was less than that of UV aging, the others were far greater than those of UV aging. Rain, wind, dust and other factors had little impact on the performance of asphalt. The aging contribution index of rain, wind, dust and other factors to the fatigue resistance of SBS-modified asphalt was above −0.7. It can be seen that the covering effect of sand blown by the wind on the asphalt surface could reduce the adverse impact of environmental factors on the fatigue resistance of asphalt. Rain, wind, dust and other factors, in addition to affecting the aging degree of the high-temperature performance of SBS-modified asphalt, did not deepen the aging of other properties.

## 4. Conclusions

In this study, natural aging samples of SBS-modified asphalt in cold and arid regions were prepared. Based on rheological parameters, the effects of natural aging on viscoelastic properties, high-temperature performance, fatigue resistance and cracking resistance at a low temperature of SBS-modified asphalt were analyzed. The quantitative evaluation index of asphalt natural aging contribution rate (*CI*i) was proposed, and the contribution rates of various environmental factors to natural aging of asphalt in cold and arid regions were analyzed. The main conclusions were as follows:

1. After natural aging, the complex modulus of asphalt was reduced, and the risk of fatigue cracking and low-temperature cracking were increased. Aging also enhanced the anti-deformation ability of asphalt and improved the high-temperature rutting resistance.
2. The aging contribution index *CI*i directly reflected the influence of different aging factors on the performance of asphalt. Among them, the thermal oxygen effect had the greatest contribution to the viscoelastic properties, high-temperature rutting resistance and low-temperature cracking resistance of SBS-modified asphalt.
3. One year of natural aging had little impact on the aging degree of asphalt, but it could still be clearly felt that the aging degree of asphalt was deepened by a large temperature difference, oxygen and UV radiation. Large temperature difference, oxygen and UV radiation pose great challenges for pavements in Northwest China.
4. The aging law of asphalt samples with different thicknesses, the aging evolution model of asphalt and the natural aging law of asphalt mixture still need to be further studied.

## Figures and Tables

**Table 1:** Basic performance indexes of SBS-modified asphalt.

Test Items		Quality Index	Detection Value
Penetration (25 °C, 100 g, 5 s)/(0.1 mm)		60~80	66
Penetration index PI not less than		−0.4	−0.1
Ductility (5 °C) not less than/cm		35	42
Softening point (R&B) not less than/°C		75	87.5
135 °C Brookfield rotational viscosity/(Pa·s)		1.8–3.0	1.91
Flash point not less than/°C		230	280
Solubility not less than/%		99	99.5
Elastic recovery (25 °C) not less than/%		85	94
Storage stability not greater than/°C		2.5	0.8
Residue after RTFOT	The quality change is not greater than/%	±1.0	−0.4
	Penetration ratio (25 °C) not less than/%	65	75
	Ductility (5 °C) not less than/cm	20	30

**Table 2:** Effect of different aging factors on SBS-modified asphalt.

Properties	Effect of Aging
Viscoelastic properties	UV aging > TO aging > Others (ω: 0.1–0.18) TO aging > UV aging > Others (ω: 0.18–100)
High-temperature performance	TO aging > UV aging > Others
Fatigue resistance	UV aging > TO aging > Others
Low-temperature cracking resistance	TO aging > UV aging > Others

## Data Availability

The study did not report any data.
